# Topographic Analysis of the Periorbital Region Including Orbicularis Oculi Muscle Based on Ultrasonography Interpretation

**DOI:** 10.1111/jocd.70004

**Published:** 2025-01-21

**Authors:** Kyu‐Lim Lee, Hee‐Jin Kim

**Affiliations:** ^1^ Division in Anatomy and Developmental Biology, Department of Oral Biology Human Identification Research Institute, BK21 FOUR Project, Yonsei University College of Dentistry Seoul South Korea; ^2^ Department of Materials Science & Engineering, College of Engineering Yonsei University Seoul South Korea

**Keywords:** orbicularis oculi muscle, periorbital region, soft tissue thickness, ultrasonography

## Abstract

**Objective:**

Ultrasonographic examination is easy, fast, safe, and used in various fields; however, its application to the facial area has been limited. Complex anatomical structures are mixed within thin, soft tissues in the facial region; therefore, understanding their structural characteristics is crucial. This study aimed to use ultrasonography to obtain information on the layered structure and soft tissue thickness of the eye area around the orbicularis oculi muscle and provide guidance for clinical practice.

**Methods:**

Healthy volunteers (33 men and 19 women; mean age: 28.4 years) underwent ultrasonography with nine reference points. The soft tissue thickness, including the orbicularis oculi muscle, was measured on monochromatic images. Ultrasonographic scans were performed at facial landmarks using linear transducers (IO8‐17, E‐CUBE15, Alpinion Medical System, Seoul, Korea), with images scanned transversely. The thickness was measured using ImageJ (National Institutes of Health, Bethesda, MD, USA).

**Results:**

The mean thickness of the orbicularis oculi muscle was 1.56 ± 0.45 mm (range 1.03–2.31 mm). The highest thickness was measured at the points VII (2.31 ± 0.68 mm) and VI (2.15 ± 0.48 mm). The mean depth of the orbicularis oculi muscle was 1.63 ± 0.62 mm (range 0.88–2.80 mm), and the most superficial point was 0.88 ± 0.99, at point VII.

**Conclusion:**

This study provides critical anatomical data that can enhance the precision of ultrasound‐guided procedures in the periorbital area, allowing clinicians to accurately target the muscle layer and soft tissue structures. By utilizing these findings, practitioners can optimize treatment effectiveness, reduce complications, and improve outcomes in cosmetic procedures such as botulinum toxin injections, filler placements, and other non‐invasive facial treatments. The detailed anatomical insights gained from this study will help bridge the gap between anatomical understanding and clinical application, promoting safer and more efficient aesthetic interventions.

## 1 Introduction

Ultrasonographic examination is easy, fast, safe, and widely used across various fields. However, because the anatomy of the face is small and complex, the use of facial ultrasonography has been relatively insignificant. Recent studies have attempted to apply ultrasonography to the facial area; however, most are related to clinical procedures, with insufficient construction of the basic ultrasonographic data of the face. Therefore, providing basic anatomical data based on ultrasonographic interpretation of the facial area is urgent. Subsequently, these data can be used to improve clinicians' understanding of ultrasonograms and develop clinical technologies for application to new procedures.

The eyes are the most prominent structure in the face and play a major role in facial expressions and expressing emotions. The orbicularis oculi muscle facilitates this role, although the periorbital region has a complex structure of various blended muscles. The orbicularis oculi muscle, a broad and flat‐shaped muscle extending to the lower eyelid and eyebrow area, plays a key role in closing the eyelids, protecting the eyeball, and shaping facial expressions. When active, it mediates the contraction of the skin of the lateral orbit, causing skin wrinkles. The complex muscle fibers of the orbicularis oculi muscle generate wrinkles in several directions, particularly noticeable in crow's feet, a common sign of facial aging [1, 2]. Wrinkles occur owing to the contraction and relaxation of facial muscles concentrated in areas linked to emotional expressions, such as the forehead, eyes, nose, and mouth [3]. These wrinkles are only noticeable during emotional expression and are absent on an expressionless face, but when the wrinkles become deeper, patients want to relieve them [4]. To improve these symptoms, botulinum toxin injections are generally performed, and safe and effective botulinum toxin injection procedures require clear information about the structure and depth of soft tissue.

In addition to recent ultrasonographic research, previous studies have presented data based on cadavers to provide information on the soft tissue around the eye. Our earlier study found that the mean distance between the lateral canthus and lateral edge of the orbicularis oculi muscle was 31 mm. Moreover, the medial and lateral bands of the orbicularis oculi muscle were observed in 64% and 54% of the population, respectively [3]. Hur et al. reported on the width of the orbicularis oculi muscle extending laterally to the upper lip and its variations [5]. Meanwhile, Piao et al. described imaging for some parts of the orbicularis oculi muscle for crow's feet treatment using ultrasound‐guided injection [6]. However, it was not sufficient to provide information for application in clinical practice.

Ultrasonography on topographic and surface anatomical landmarks and the internal structure of the orbicularis oculi muscle remains limited. Further, delicate changes in facial expression due to muscle imbalance can occur after botulinum neurotoxin (BoNT) injection despite precise treatment guidelines. The lateral fiber region of the orbicularis oculi muscle, which extends to the upper lip without bone attachment, is prone to wrinkles and requires additional treatment with BoNT injections [5]. However, no study has been conducted to investigate ultrasound interpretation guidelines for soft tissue structures in the periorbital area, including detailed depth information of the orbicularis oculi muscle. Hence, this study aimed to elucidate the anatomical structure and soft tissue thickness of the periorbital region using ultrasonography to provide clinical guidance for future explorations.

## 2 Methods

Fifty‐two healthy volunteers (33 men, 19 women; mean age: 28.4 years; age range: 23–40 years) were recruited for ultrasonographic examination. Before conducting the test, all volunteers were given a detailed explanation of the purpose of the study, all methods, and possible risks, and were informed that they could stop the experiment at any time. Written informed consent was obtained from all participants, and all procedures complied with the guidelines of the Declaration of Helsinki and were approved by the Ethics Committee of Yonsei University College of Dentistry (IRB No. 2–2024‐0006).

The volunteers were positioned in a semi‐supine position for the ultrasonographic examination. Seven facial landmarks were identified and expressed on the skin surface by using a surgical pen. First, the overall position of the orbital rim was confirmed via palpation. Next, vertical lines passing through the medial, mid‐pupillary, and lateral canthus were established. Nine landmarks were set, including eight points where the vertical line met the superior and inferior orbital rims and the lateral orbital rim at the height of the lateral canthus. Each point was numbered clockwise from I to IX based on the intersection between the vertical line passing through the medial canthus and the superior orbital rims. Ultrasonograms were acquired vertically concerning the orbital rim, the measurement reference point. During the ultrasonographic imaging, participants were asked to make facial expressions such as raising their eyebrows or squinting their eyes to identify anatomical structures better.

Ultrasonographic examinations were performed using a real‐time two‐dimensional ultrasonographic scanner (E‐Cube 15, ALPINION Medical Systems, Seoul, Korea) equipped with a 30 mm wide linear array transducer (8.0–17.0 MHz; IO8‐17 High‐Frequency Hockey Stick, Seoul, Korea). A non‐irritating ultrasonographic gel (Sono Jelly, Meditop Corporation, Yongin, Korea) was applied at each measurement point. Proper care was taken to ensure that mechanical pressure from the transducer did not alter soft tissue properties. This was achieved by applying the thick ultrasonographic gel at each point and ensuring that the transducer did not touch the skin surface. Two examiners conducted measurements at each reference point twice using ultrasonography, and the average value was presented for analysis.

Ultrasonographic morphological analysis of the soft tissue of the periorbital region, including the orbicularis oculi muscle, was performed to generate monochromatic images. ImageJ image analysis software (National Institutes of Health, Bethesda, MD, USA) was used to measure soft tissue thickness at a designated point.

## 3 Results

**FIGURE 1:**
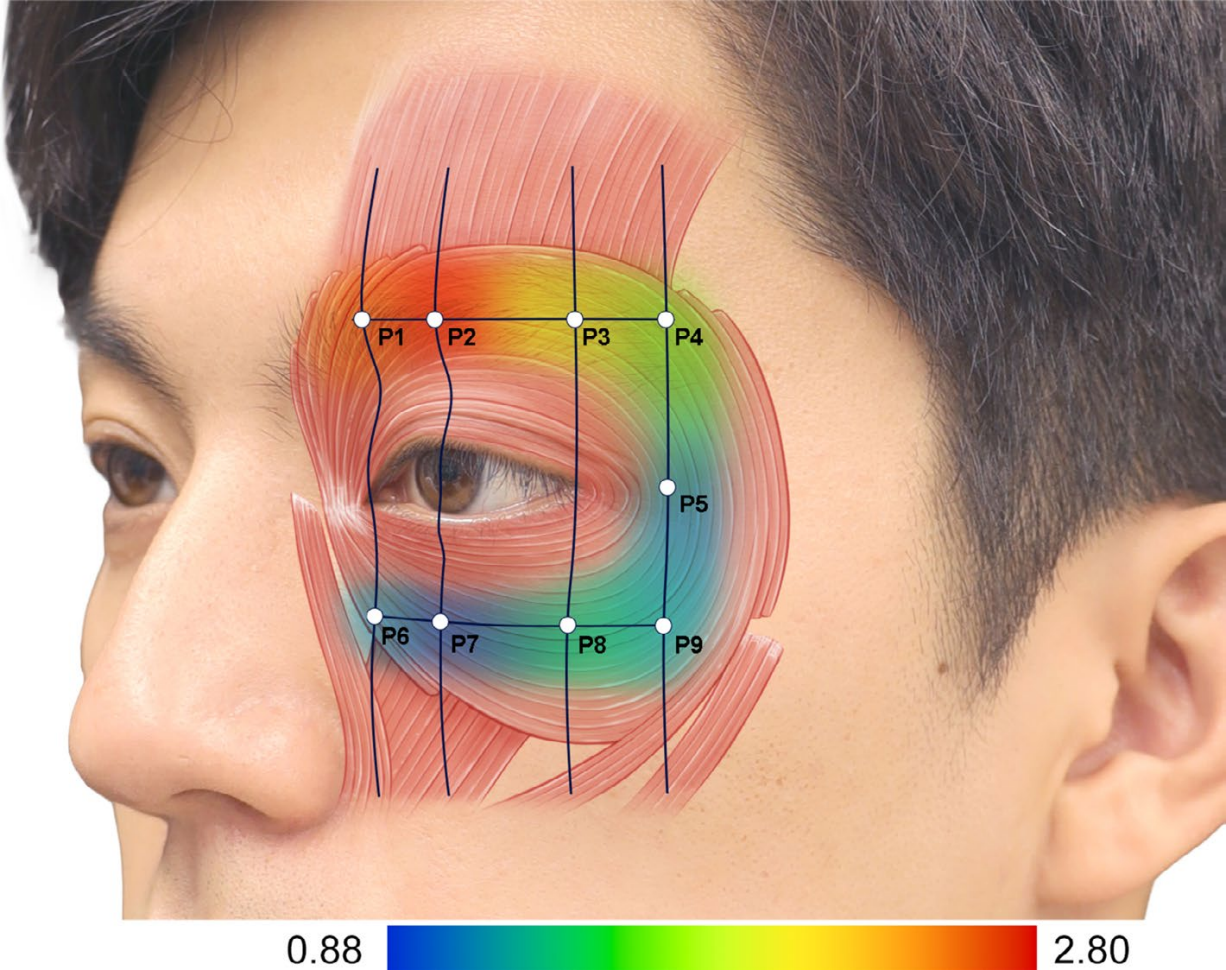
Illustration representing the depth of the orbicularis oculi muscle from the skin surface of the periorbital region at each measurement point.

The soft tissue in the periorbital area was divided into various layers, which are the skin, subcutaneous tissue, muscle, and loose connective tissue. The orbicularis oculi muscle appeared hypoechoic deep in the skin, and the fatty tissue appeared hyperechoic. Characteristic fatty tissues, such as the retro‐orbicularis oculi fat (ROOF), sub‐orbicularis oculi fat (SOOF), and inferior orbital fat, were observed around the periorbital region. The detailed depths and thicknesses of the skin, subcutaneous tissue, orbicularis oculi muscle, and total soft tissue thickness are outlined in Table 1 and Figure 1 In each case, the muscle layer was thicker than the other layers, making it easier to identify. Orbicularis oculi muscle was located at a mean depth of 1.63 ± 0.62 mm (range, 0.88–2.80 mm) from the skin surface and had a mean thickness of 1.56 ± 0.45 mm (range, 1.03–2.31 mm).

**TABLE 1:** Soft tissue thicknesses and depth of muscular structure at the measurement points.

	Point 1	Point 2	Point 3	Point 4	Point 5	Point 6	Point 7	Point 8	Point 9
Skin	1.28 ± 0.23	1.17 ± 0.22	1.12 ± 0.23	1.11 ± 0.22	0.85 ± 0.16	0.91 ± 0.15	0.88 ± 0.20	1.06 ± 0.22	1.14 ± 0.14
Subcutaneous tissue	1.40 ± 0.44	2.01 ± 0.56	1.35 ± 0.40	1.22 ± 0.38	0.81 ± 0.25	1.13 ± 0.22	0.98 ± 0.32	1.35 ± 0.21	1.55 ± 0.36
Muscle (orbicularis oculi muscle)	1.06 ± 0.23	1.44 ± 0.25	1.23 ± 0.30	1.17 ± 0.29	1.03 ± 0.27	2.15 ± 0.48	2.31 ± 0.68	1.90 ± 0.33	1.74 ± 0.31
Depth to orbicularis oculi muscle	2.46 ± 0.89	2.80 ± 1.18	1.89 ± 1.13	1.80 ± 1.04	1.11 ± 0.83	1.12 ± 1.04	0.88 ± 0.99	1.47 ± 1.21	1.16 ± 1.36
Total thickness (surface‐bone)	6.63 ± 0.97	6.35 ± 0.97	5.27 ± 0.95	4.86 ± 0.80	4.29 ± 0.81	4.87 ± 0.69	5.28 ± 1.04	6.02 ± 0.92	5.82 ± 0.66

*Note:* Data are presented as the mean ± standard deviation in millimeters.

**FIGURE 2:**
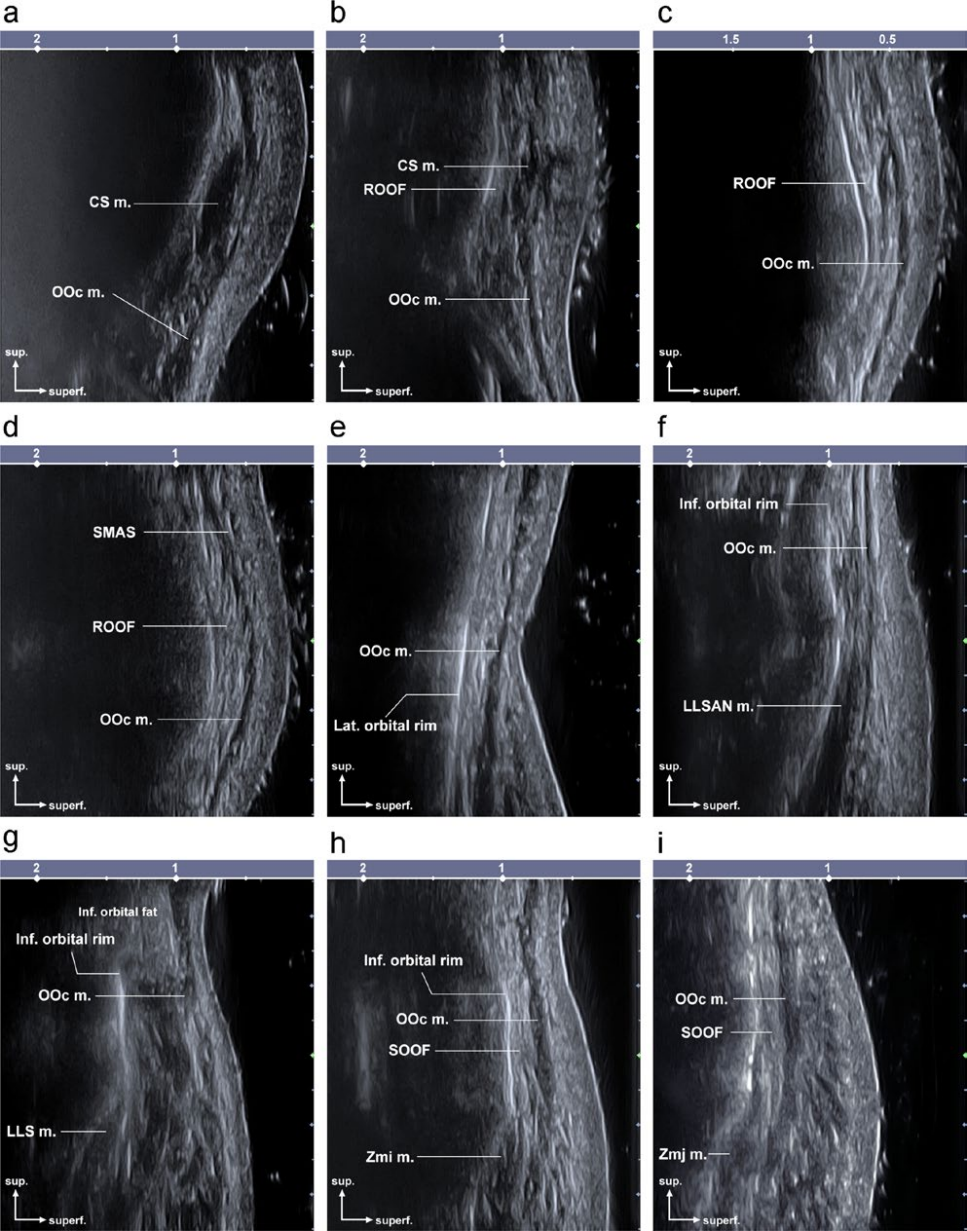
Ultrasonogram at reference points I–IX of the periorbital regions. (a), (b), (c), (d), (e), (f), (g), (h), (i); CS, corrugator supercilii muscle; LLS m., levator labii superioris muscle; LLSAN m., levator labii superioris alaeque nasi muscle; OOc m., Orbicularis Oculi muscle; ROOF, retro‐orbicularis oculi fat; SOOF, sub‐orbicularis oculi fat; Zmi m., zygomaticus minor muscle; ZMj m. zygomaticus major muscle; sup., superior; superf., superficial.

The main anatomical structures observed at each point are shown in Figure 2.

Point I: The orbicularis oculi muscle was identified as very thin. The corrugator supercilii muscle was observed deep in the orbicularis oculi muscle.

Point II: The orbicularis oculi, corrugator supercilii muscle, and ROOF were observed when the measurement was performed at the superior orbital rim point.

Point III: Observation at the superior orbital rim point revealed the orbicularis oculi muscle and ROOF.

Point IV: Observation at the superior orbital rim point revealed the orbicularis oculi muscle, ROOF, and superficial musculoaponeurotic system.

Point V: The orbicularis oculi muscle was visible, and the lateral orbital rim was easily identified.

Point VI: The orbicularis oculi and levator labii superioris alaeque nasi muscle were observed. The orbicularis oculi muscle fibers did not have a flat shape like other points, and the medial band of the orbicularis oculi muscle was confirmed to be round.

Point VII: Observation at the inferior orbital rim point revealed the orbicularis oculi and levator labii superioris muscles originating from the bone. In addition, the inferior orbital fat was voluminous.

Point VIII: The orbicularis oculi muscle and SOOF were located between the bony surface and the orbicularis oculi muscle. Additionally, the bony origin of the zygomaticus minor muscle was observed.

Point IX: The orbicularis oculi muscle was visible, like the results from point VIII. The SOOF was located between the bone and orbicularis oculi muscle. Additionally, the bony origin of the zygomaticus minor muscle was observed.

## 4 Discussion

Despite the various advantages of ultrasonography, its application in assessing the head and neck region is limited. However, recent studies have illustrated its potential applications by providing guidelines for safe and effective procedures and identifying crucial anatomical structures [1, 6, 7, 8, 9]. Facial expression muscles, distinct from skeletal muscles, present a unique challenge owing to their location beneath the skin, origin from the bone, and insertion into the skin. The variable thickness of facial expression muscles emphasizes the need to elucidate soft tissue structures in the facial area for clinical practice. The orbicularis oculi muscle is located across the upper and mid‐facial regions. Facial expression muscles are particularly thin and have a three‐dimensional shape, with thick band‐shaped tendons formed on their inner and lateral sides. The orbicularis oculi muscle, which plays an important role in eyelid closure, can be divided into four parts: orbital, palpebral, lateral, and medial bands. The muscle then surrounds and inserts into the lateral and medial canthal tendons and partially inserts into the frontalis muscle, procerus muscle, corrugator supercilii muscle, and skin [4].

The complex muscle fibers of the orbicularis oculi muscle produce wrinkles in various directions, depending on their location [3]. Generally, subdermal or intradermal injection is recommended when wrinkles appear around the eyes. Previous studies have suggested that the skin and facial muscles are the two main causative factors for wrinkle formation, and paralysis of facial muscles has been suggested as an effective treatment [10]. Furthermore, various aesthetic treatments, such as laser, high‐intensity focused ultrasonography, filler, and BoNT treatments, have been proposed to reduce the obvious appearance of wrinkles [6, 11]. BoNT injection into the lateral orbital portion eases or prevents lateral canthal rhytids, known as the crow's feet. However, the associated anatomy has not been adequately described, and this lack of information challenges treatment approaches and causes side effects [12]. Previous ultrasonographic studies have reported changes in the orbicularis oculi muscle thickness when frowning; however, only at limited points. The results of a previous ultrasonographic study showed that the lateral part of the orbicularis oculi muscle became thicker when relaxed and contracted strongly and moved medially during dynamic eyelid closure [6]. Imhof and Kuhne showed that the microdroplet technique for administering BoNT‐A improved wrinkle formation in the lower crow's feet, indicating that the area is an effective target area, underscoring the need for cautious targeting for effective treatment. Local muscle paralysis is an effective intervention for wrinkle formation, which is attributed to the skin and facial expression muscles [13].

The orbicularis oculi muscle is crucial to various procedures, including composite rhytidectomy and eyelid blepharoplasty, and addressing specific concerns, such as crows' feet and palpebromalar grooves [6]. Therefore, understanding its three‐dimensional anatomical structure is essential to performing these procedures. Ultrasonographic guidance for diagnostic and therapeutic injections faces challenges in imaging facial expression muscles owing to their unique characteristics [1, 14, 15]. Unlike typical skeletal muscles, facial muscles lack a fascial sheath, making identifying their muscle boundaries challenging. Their thin and flat nature further complicates the identification of the overlapping muscle groups. Despite these challenges, awareness of the morphological factors and variations in facial muscles contributes to developing effective ultrasound‐guided injection techniques [1].

Our results showed that the mean thickness of the orbicularis oculi muscle was 1.56 ± 0.45 mm (range, 1.03–2.31 mm). The highest thickness was observed at points VII and VI at 2.31 ± 0.68 mm and 2.15 ± 0.48 mm, respectively. The mean depth of the orbicularis oculi muscle was 1.63 ± 0.62 mm (range, 0.88–2.80 mm), and the most superficial point was 0.88 ± 0.99, at point VII. According to previous research, no significant differences exist between the sexes. Therefore, differences between sexes were not reported in the results of this study [1, 16]. In addition to the orbicularis oculi, the corrugator supercilii, levator labii superioris alaeque nasi, and zygomaticus minor were located at the measured points. Clinically important fat structures, such as the ROOF, SOOF, and inferior orbital fat, were also observed. Clinicians can perform effective and safe procedures for various treatments around the eyes based on these suggested points and information on soft tissue thickness.

Ultrasound‐guided injection is an excellent method for safe and effective noninvasive treatment [4]. A comparative study between blind and ultrasound‐guided BoNT injection demonstrated increased safety and reliability with ultrasound guidance, emphasizing its advantage [17]. Using ultrasonography, clinicians can easily identify soft tissue layers and accurately identify target points to avoid complications. Therefore, this study provided basic anatomical information that clinicians can apply to various injectable treatments in the periorbital region.

This study aimed to eliminate deformation while observing the layered structure and measuring the soft tissue thickness by applying sufficient gel. This approach allowed us to obtain ultrasonograms without pressure between the transducer and soft tissue. In this study, the probe was positioned with its center aligned to the orbital rim, ensuring consistent imaging landmarks. Given the 2 cm length of the probe, both the orbital and palpebral parts of the orbicularis oculi muscle were captured within the scanned images. Although it is challenging to distinctly delineate these subregions on ultrasonographic images, the observed muscle thickness appeared relatively consistent between the upper and lower sections. Rather than isolating individual parts, this study focused on providing a comprehensive understanding of the overall muscle structure, which we believe is more relevant to clinical applications. Figures 1 and 2 illustrate the captured regions and their anatomical characteristics, offering practical insights for clinicians.

Despite the strengths of this sstudy, there are limitations. The focus was primarily on the layered structure and thickness of the periorbital region without detailed analysis of the nervous and arterial distributions, which are crucial for avoiding procedural complications. Additionally, the study population consisted of relatively young participants, necessitating further research on older adults to enhance the generalizability of the findings. Future studies should also explore three‐dimensional ultrasonographic techniques to provide a more comprehensive understanding of the facial anatomy, which could lead to the development of improved clinical guidelines.

## 5 Conclusion

Based on the results of this study, clinicians can use ultrasound devices to accurately identify soft tissue layers in the periorbital area and differentiate anatomical structures crucial for cosmetic procedures. This study provides key data on the orbicularis oculi muscle, with an average thickness of 1.56 mm and a depth ranging from 0.88 to 2.80 mm, aiding in the precise targeting of muscle layers during ultrasound‐guided injections. These findings support the safe, effective, and efficient application of various injection procedures, such as botulinum toxin treatments, thereby enhancing clinical outcomes in aesthetic dermatology.

## Author Contributions

All authors were well‐informed of the WMA Declaration of Helsinki‐Ethical Principles for Medical Research Involving Human Subjects and confirmed that this study firmly fulfilled the declaration. None of the authors has financial or private relationships with commercial, academic, or political organizations or people that may have improperly influenced this research. Overall planning of the research, data acquisition, analysis and interpretation, and major drafting and revision of manuscript submission was done by K.‐L.L.; providing the anatomical and clinical opinion for conception, overall organization, and direct supervision of the research was undertaken by H.‐J.K.

## Ethics Statement

Written informed consent was obtained from all participants, and all procedures complied with the guidelines of the Declaration of Helsinki and were approved by the Ethics Committee of Yonsei University College of Dentistry (IRB No. 2–2024‐0006).

## Conflicts of Interest

The authors declare no conflicts of interest.

## Data Availability

The data presented are available on request from the corresponding author.
