# Correction to “Topographic Analysis of the Periorbital Region Including Orbicularis Oculi Muscle Based on Ultrasonography Interpretation”

**DOI:** 10.1111/jocd.70065

**Published:** 2025-02-20

K.‐L. Lee and H.‐J. Kim, “Topographic Analysis of the Periorbital Region Including Orbicularis Oculi Muscle Based on Ultrasonography Interpretation.” *Journal of Cosmetic Dermatology* 24, no. 1 (2025): e70004, https://doi.org/10.1111/jocd.70004.

In the published article, the corresponding author's email address was incorrectly listed as “hjk887@yuhs.ac”. The correct email address should read: “hjk776@yuhs.ac”.

We apologize for this error.

